# TAEO-A Thermal Aware & Energy Optimized Routing Protocol for Wireless Body Area Networks

**DOI:** 10.3390/s19153275

**Published:** 2019-07-25

**Authors:** Mohsin Javed, Ghufran Ahmed, Danish Mahmood, Mohsin Raza, Kamran Ali, Masood Ur-Rehman

**Affiliations:** 1 Department of Computer Science, COMSATS University Islamabad (CUI), Islamabad 44000, Pakistan; 2 Department of Computer Science, Shaheed Zulfiqar Ali Bhutto Institute of Science and Technology (SZABIST), Islamabad 44000, Pakistan; 3 Faculty of Science and Technology, Middlesex University, The Burroughs, Hendon, London NW4 4BT, UK; 4 School of Computer Science and Electronic Engineering, University of Essex, Wivenhoe Park, Colchester CO4 3SQ, UK

**Keywords:** energy efficient, hot spot, power dissipation, thermal aware, WBAN

## Abstract

Wireless Body Area Networks (WBANs) are in the spotlight of researchers and engineering industries due to many applications. Remote health monitoring for general as well as military purposes where tiny sensors are attached or implanted inside the skin of the body to sense the required attribute is particularly prominent. To seamlessly accomplish this procedure, there are various challenges, out of which temperature control to reduce thermal effects and optimum power consumption to reduce energy wastage are placed at the highest priority. Regular and consistent operation of a sensor node for a long-time result in a rising of the temperature of respective tissues, where it is attached or implanted. This temperature rise has harmful effects on human tissues, which may lead to the tissue damage. In this paper, a Temperate Aware and Energy Optimized (TAEO) routing protocol is proposed that not only deals with the thermal aspects and hot spot problem, but also extends the stability and lifetime of a network. Analytical simulations are conducted, and the results depict better performance in terms of the network lifetime, throughput, energy preservation, and temperature control with respect to state of the art WBAN protocols.

## 1. Introduction

Advancement in Information and Communication Technology (ICT) has revolutionized all domains of applied sciences. One such domain that has gained much attention of the research arena, as well as engineering industries, over last two decades is Smart Health using Wireless Body Area Networks (WBANs). WBAN has a potential to solve social problems, like efficient use of limited medical infrastructure with respect to population, raising aging population, and over wrought facilities. Hence, there is a dire need for the prevailing conditions to attract general public towards WBAN technology to address the aforementioned problems.

Recent trends and enhancements in communication and sensory technology have led to the further optimization of WBAN. WBAN is a type of Sensor Network, which has numerous applications both for medical and non-medical purposes [1,2], like in defense activities, by physicians for remote health monitoring, for coaches and players in sports, etc. The generic purpose of WBAN is the automation of certain processes for the ease of people and, consequently, bringing improvements in their lifestyle. Therefore, the technological significance of WBAN is eminent from its day-to-day applications.

**Figure 1:**
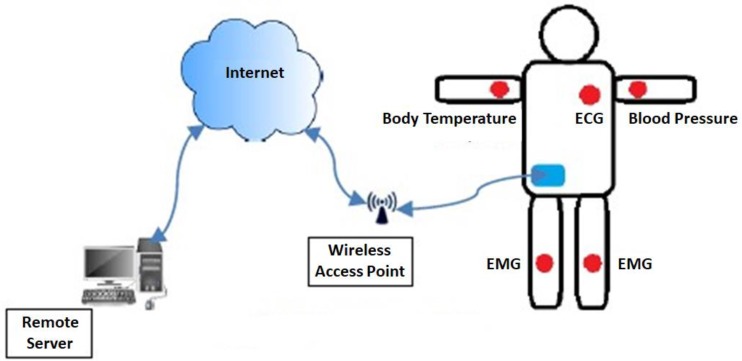
Wireless Body Area Networks (WBAN) basic architecture depicting deployment of sensors for gathering patient’s data and submitting it to a remote server via the internet.

WBAN constitutes a set of miniature sensors, like Electrocardiogram (ECG) and Electromyography (EMG), which are implanted inside the human body or placed outside the skin; Figure 1 shows a basic WBAN architecture. These nodes are integrated into the exchange of information. Each sensor collects the associated data from its surrounding and processes it for onward submission to the intended medical rep or physician. The adoption of technology always imposes certain challenges; similar is the case with WBAN. Aside from its proved assistance to both patients and doctors, some aspects of WBAN need to be controlled for its practical implementation.

In addition to the saved energy through intelligent design of electric circuits of wireless sensor nodes, energy efficient routing plays a vital role in preserving the energy of these nodes. Moreover, another vital problem in such networks is the thermal dissipation of nodes, which is harmful for human tissues. In WBAN, a node is declared as a hot-spot node when its temperature is raised to an extent where it can damage human tissues. This is a safety hazard and it is not acceptable in WBAN implementation. Therefore, the temperature of a hot-spot node needs to be intelligently managed for a hazard-free deployment of WBAN. There are various factors that cause the temperature rise of the node. Two primary sources [3] are:

- EM transmission and
- power consumption within internal circuitry.

### Problem Statement and Contribution

In WBAN, a node communicates with its neighbors or sink using radio frequency (RF) signals. A transceiver in a sensor device is responsible for the communication of data through electromagnetic waves. This radio frequency transmission dissipates heat, which is absorbed by tissues, and consequently raises the temperature. The second factor causing temperature rise is the internal circuitry of a sensor node that generates heat while processing the collected data. The amount of heat that is generated by a node circuitry is dependent on its architecture. There is a need to control a node from dissipating excessive heat. Therefore, heat that is generated by the sensor nodes needs to be controlled for effective implementation of WBAN. An increase in the thermal dissipation of a sensor node due to the transmission of packets is one core safety issue in WBAN [4]. This is the major theme of the proposed protocol i.e. to keep the heated node away from the transmission process until its temperature revamped in order to provide a safe WBAN system for the health monitoring of patients. As discussed earlier, heat dissipation mainly depends on electromagnetic communication and the internal circuitry of a node. Therefore, this issue needs to be intelligently addressed in a practical implementation of WBAN technology. Otherwise, an increase in the temperature of a sensor node to a menacing level results in the damage of human tissues or DNA. In WBAN, this is also known as hot-spot node problem. 

In this work, a novel routing protocol for WBAN is proposed, which provides a solution to the following two problems:

- effective detection and avoidance of a hot-spot node based on Specific Absorption Rate (SAR) and
- enhancement in stability period and network lifetime.

The proposed routing protocol has out classed two state-of-the-art routing protocols in terms of stability period, throughput and residual energy. Moreover, an analysis of the ratio of heated and normal nodes is also conducted.

Rest of the paper is organized, as follows: Section 2 discusses the related work. Section 3 presents the system model. The proposed methodology is described in Section 4. Section 5 presents the results and discussion. Finally, Section 6 concludes the paper.

## 2. Related Work

The researchers have done immense work to bring the continual advancement of WBAN technology. This section explains the notable work of authors on WBAN.

In [3], the authors proposed the Thermal Aware Routing Algorithm (TARA), which is a pioneer protocol in the thermal-aware WBAN. The work before TARA did not encompass the effects of temperature rise on the human body. RF communication and internal circuitry of the sensor are two major sources of heat generation. On the base of these sources, the hot-spots are identified. Data sensing, processing, and transmitting activate ultimately increase the temperature of a node. In TARA, a node monitors the transmission of packets by its neighbors. On this base of this data, it estimates the temperature rise of a node. When the estimated temperature reaches a threshold, the node is marked as a hot-spot. The traffic towards the hot-Spot node is withdrawn and then returned to the previous node in situations where all of the neighbors are hot-spot. The packet withdrawal strategy adds up the overhead of rerouting and leads to overall transmission delay. Power consumption is also increased due to additional rerouting.

In [5], Takahashi proposed the Least Temperature Rise (LTR) scheme to address the shortcomings of TARA. The idea of the coolest neighbor is introduced, i.e. lowest temperature node is opted as a data forwarder. The protocol implemented a counter for the number of hops to limit the stray packet and to avoid looping. When the limit is reached, the packet is eliminated or dropped from the network. A list of recently visited nodes is maintained to optimize the node selection process. If the coolest node is in that list, then the node with the second lowest temperature is selected. As LTR is based on a greedy approach, the packets undergo longer delays to reach destination, thus undermining the reliability of communication. In the same paper, the author proposed ALTR, which is an improved version of LTR. In this scheme, the Adaptive Threshold is used and, when the hop count limit is reached, the packet is not dropped or discarded, but it is forwarded to the destination using the shortest-path algorithm. The protocol successfully reduced the transmission delay. However, when a shortest-path algorithm is applied, the protocol allows the traffic through a hot-spot node.

In the same paper [5], the Least Total Route Temperature (LTRT) protocol works by pre-identification of routes from source to destination, and thereby selecting the path with the least total route. In LTRT, the temperature of a sensor is treated as its weight and the total route from source to destination is calculated and the minimum cost total route is opted for communication.

HPR [6] uses the Shortest-Hop algorithm for transmission of packets to the destination. It works by defining a temperature threshold and allowing transmission if the nodes temperature is below the specified limit. If a nodes temperature surpasses the threshold, then the packets are rerouted to a neighbor with the lowest temperature. The exchange of information related to the node’s heat in between nodes for hot-spot detection is a major overhead. 

The Thermal-Aware Shortest Hop Routing (TSHR) scheme for in-vivo implementation of WBAN is presented in [7]. Tabandeh and Jahed presented a novel architecture with a two-phase implementation approach. The configuration of nodes and the exchange of necessary information are managed in the first phase. Whereas, the second phase implements the routing scheme and the packets are forwarded on the path with a minimum number of hops to the destination. In TSHR, two temperature thresholds are specified. The first limit is for maximum acceptable temperature by a node. The second limit is dynamic and established on the base of a neighbor’s temperature. Afterward, the node inspects and, if neighbor’s temperature exceeds the preset dynamic value, it is declared as a hot spot.

In [8], Mobile-ATTEMPT (M-ATTEMPT) is proposed, which is an extension of ATTEMPT routing scheme. M-ATTEMPT is a thermal-aware WBAN protocol with efficient mobility support. It works by defining the zones with levels, depicting a parent-child relation. When a node disconnects and enters another zone, it requests the respective parent node for becoming a member. The parent node only accepts the request if the child node count is less than three.

The authors in [9] proposed a novel routing scheme i.e., Energy Efficient Thermal and Power Aware (ETPA), which is focused to provide a thermal-aware and low power consumption implementation of WBAN. Routes are defined on the base of communication expense, which is calculated when considering the energy level, temperature, and signal strength of the neighbors. ETPA works by establishing a four-frame cycle, in which every sensor exchanges its current heat- value and energy level with all other nodes. Afterward, each node in the cycle calculates the transmission cost based on the signal power of the received signal from an adjacent node. The low-cost route is opted for transmission. ETPA has managed the temperature of a sensor by diffusing it amongst other nodes, and thus results in improved lifetime due to lowered energy depletion.

In [10], the authors apprised that the increase or decrease in Specific Absorption Rate (SAR) value primarily depends on the strength of the surrounding electromagnetic field. Other parameters, such as the characteristics of tissue, its dimensions, including size, frequency, and exposure duration to radiations forms the basis of good SAR estimates. SAR estimates and the associated parameters differ, depending on the type of tissue, like brain, muscles, or nerve.

The authors of [11] performed a lot of experimental work to identify safe SAR boundaries. There work proved to be highly beneficial in the development of safe wireless systems. SAR values below 0.02 W/kg are proven to be safe. Experiments that were performed using high SAR values illustrate that the 1.0–1.2 W/kg SAR range has shown the disparaging impact on the chromosome, and consequently damaged the DNA. In [12], Bag and Bissiouni established an effective routing scheme for WBAN to cater to heated node avoidance and successfully reduced the average delay. The transmission path is determined on the base of a number of hops and least hop count route is selected. Rerouting is required in the case of a hot-spot in the finalized route.

The authors in [4] proposed a novel routing model comprising of upper and lower temperature thresholds. When the node temperature attains the lower threshold, the traffic is controlled in order to decrease the rate of temperature rise. Upon the surpassing of the upper limit, the node is marked as a heated node and all of its traffic is re-routed to avoid a further rise in temperature.

In [13], a WBAN protocol that is based on single-hop and multi-hop implementation for critical and non-critical transmission is presented. It works by selecting a data aggregator and forwarder on the base of a cost function. The minimum cost node is opted to perform the necessary transmission to directly linked node or to the sink. The result shows the effectiveness of the proposed scheme in comparison to M-ATTEMPT protocol.

In [14], Monowar and Bajaber developed a routing scheme that focused on the traffic rate of a sensor node. Increased transmission rate elevates the chance of congestion in the network and, consequently, disrupts the Quality of Service (QoS) requirements necessary for a reliable system. This high rate of traffic and network congestion gives rise to the temperature of a node. Congestion is estimated by using queue occupancy, whereas the traffic intensity also provides vital information to measure the level of congestion. 

Authors in [15] proposed a routing scheme focused on efficient and reliable delivery of critical data in the specified duration. The protocol works by segregating critical from non-critical data and it addresses the temperature management issue.

In [16], the authors proposed RE-ATTEMPT an extension of M-ATTEMPT protocol to overcome its shortcomings. The M^2^E^2^ protocol proposed in [17] is focused on low energy consumption and improved network lifetime. The protocol works by selecting parent nodes that are sensors with comparatively high data rate placed closer to the sink. The first level and second level child nodes are identified, depending on the hierarchy in which they are linked or transmits data. The protocol works in two modes. When at home, a node on the human body is connected to the Home nodes that perform the required routing using single-hop communication. In case of non-availability of Home-Signal, the routing tables are maintained by nodes that were implanted on the human body and use multi-hop for the transmission of packets.

To solve thermal problem of WBAN, K.S.Kathe and U.A.Deshpande proposed a thermal aware routing algorithm [18]. In the proposed algorithm, the packets are transmitted on the basis of assigned priority. The objectives of this work are to maintain the temperature of the sensor nodes, reduce the packet delay, and increase packet arrival ratio. 

An Adaptive Thermal-Aware Routing (ATAR) protocol is proposed in [19] on the basis of adaptive route diversion, avoiding the heated nodes eliminating the chances of the hotspot node. The multi ring routing approach is applied in this protocol that finds an alternate optimum route.

In [20], a secondary base station is selected that helps to reduce the temperature of the neighbor nodes of master base station, as these neighbor nodes will not be a part of the new data routes from the sensing odes to the base station. In this time, the sensor node will only transmit the priority packets to the secondary base stations. Accordingly, the network load reduces by this way, and at this time the nodes have the opportunity to cool down. However, this protocol only works in the network of low and intermediate traffic and it increases the delay during high traffic. Table 1 presents the summary of commendable work that was conducted in previous years when considering thermal and energy aware routing for WBANs.

## 3. System Model

In the following section, the system model of the proposed routing protocol is presented.

### 3.1. Radio Model

In WBAN, the radio frequency model for packet transmission plays a pivotal role and it needs to be intelligently selected when considering the design requirements. TAEO is based on the first order electromagnetic transmission scheme [21]. Equations (1)–(3) estimate the power consumed by a sensor during transmission (transmit or receive).

$$P_{Tx}\;\left(N,l\right)=\;P_{Circuit-Tx}\left(N\right)+\;P_{Tx-Amp}\left(N,l\right)$$

(1) $$P_{Tx}\;\left(N,l\right)=\;P_{Circuit-Tx}.N.l^{2}$$

$$P_{Rx}\;\left(N\right)=\;P_{Circuit-Rx}\left(N\right)$$

(2) $$P_{Rx}\;\left(N\right)=\;P_{Circuit-Rx}.N$$

where, *l* is the distance between transmitter and receiver sensor. *l*^2^ indicates the power that was consumed by the transmission channel and $P_{Tx}$ is the energy utilized to send a packet from transmitter to receiver node. It is sum of the power that is consumed by the internal circuit $P_{Circuit-Tx}$ and signal amplifier $P_{Tx-Amp}$.

$P_{Rx}$ shows the power consumption of the sensor on the receiving side, and it depends on the energy that is utilized by internal circuitry only i.e., $P_{Circuit-Rx}$, as no signal amplification is required.

In WBAN, the human body also adds some attenuation in the signal. Therefore, Equation (1) includes coefficient *c* depicting the path loss:

(3) $$P_{Tx}\;\left(N,l\right)=\;P_{Circuit-Tx}*N+\;P_{Tx-Amp}.N.c.l^{2}$$

The equations that are derived above also highlights the importance of hardware selection in a routing protocol. In TAEO, the Nordic nRF 2401A sensor device is opted due to its low power consumption, which has been further improved by effectively controlling the transmission power.

### 3.2. Thermal Model

The Specific Absorption Rate (SAR) is an effective tool to identify the rate at which the heat is absorbed by tissue per unit of its mass. It estimates the heat that is absorbed due to RF waves exposure for a specified duration and for a specific device. Using SAR, limits can be identified for a sensor node to curtail the dissipation of heat accordingly. SAR depicts the probable biological concern on tissues due to RF communication. For mobile phones, the Federal Communication Commission (FCC) has provided a safe SAR range for adherence [22,23]. These are highly beneficial in designing wireless communication systems. The surpassing of SAR limits leads to damage to the tissues [24]. SAR can be evaluated through the Equation (4):

(4) $$SAR=\left(\frac{\sigma E^{2}}{\rho }\right)$$

where, value gives conductivity of the tissue is represented by σ, ρ is the density, and E is the induced electromagnetic field indicating the strength. 

## 4. Proposed Methodology-TAEO

As discussed earlier, the proposed routing protocol addresses two-core implementation issues associated with in-vivo sensors:

1. effective detection and avoidance of a Hot-Spot node based on SAR and
2. enhancement in stability period and network lifetime.

**Figure 2:**
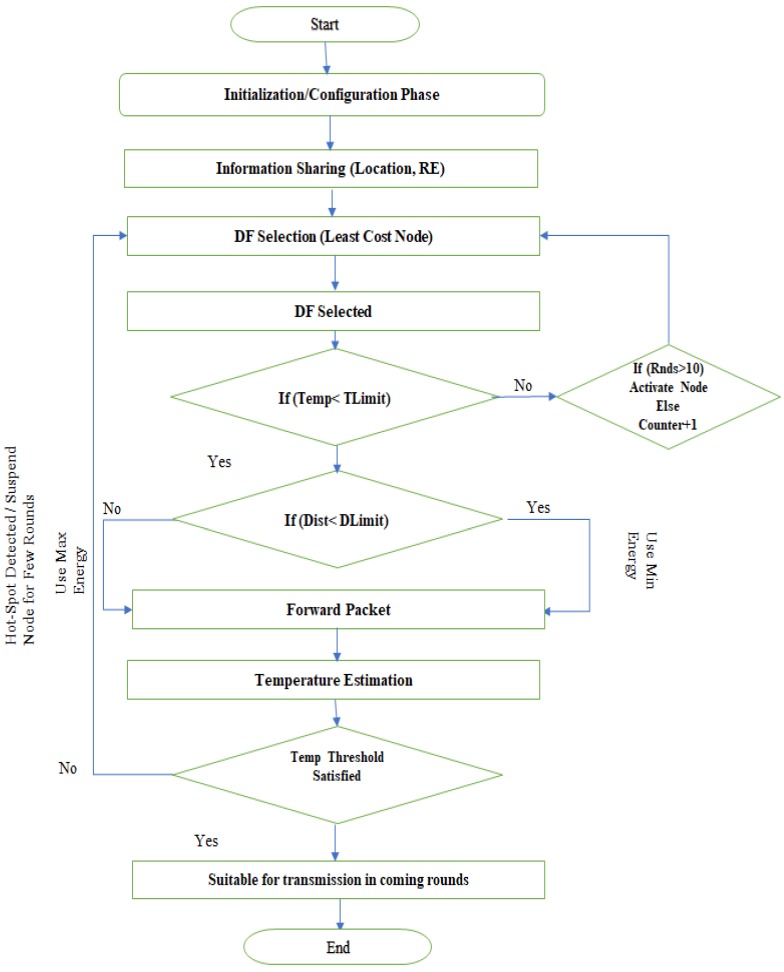
Operational Flow Diagram.

Figure 2 presents the operational flow diagram of the proposed methodology. The model is segregated into three phases, as discussed in the following sections:

### 4.1. Initialization

In this phase, each node broadcasts a packet containing the coordinates to its location, energy level, heat value, and the node identifier. In this way, all of the nodes are updated with the location of their neighbors and related information. Sink then broadcast a packet and disseminates its location to all of the nodes on the body. The temperature threshold is a value where there is a chance of tissue damage. This becomes two-fold when the temperature value exceeds from the threshold value. The most important aspect in WBAN is to avoid tissue damage as the heat that is generated by the node’s circuitry and antenna could cause damage to the human tissue [25]. The fact that the human body has a thermoregulatory mechanism to balance the heat around the body does not mitigate the issue of tissue damage when the heat received rate is larger than the thermoregulatory mechanism rate [25]. This issue can be resolved by avoiding those implantable nodes in the routing path that have been heavily utilized and may cause a risk of tissue damage. A metric, which is called Specific Absorption Rate (SAR), can be used to measure the rate at which energy is absorbed by tissues when exposed to an electromagnetic field. Experiments show that exposure to an SAR of 8 W/Kg in any gram of tissue in the head or torso for 15 min may have a significant risk of tissue damage [26]. Even with more modest heating, organs that are especially sensitive to any temperature increase due to a lack of blood flow to them are prone to thermal damage (e.g., lens cataracts [27]). Researchers have also expressed concern regarding the heating of the eye by examining the SAR and the temperature of the eye when exposed to a Wireless LAN [28] or Infrared [29] radiation. In [30], a detailed description about the thermal effects of bioimplants has been presented.

When considering the above discussion, the threshold of the parameter Temp used in Figure 2 is set according to the application requirement where the system is going to be used. It would be different when the sensors are places in the head instead of any other part of the body.

### 4.2. Routing

The proposed routing protocol is based on a multi-hop schema and it works by selecting a Data Forwarder (DF), who is responsible for the collection of packets from surrounding nodes and thereby forwarding them to sink for onward submission to a remote server. 

A node with less temperature and distance from the sink along with higher residual energy is selected as DF. The temperature of DF is estimated, and is only allowed for transmission if it is below the specified threshold; otherwise, the respective DF is suspended for upcoming few rounds, until its temperature is revamped to normal. As a multi-hop routing protocol, a neighbor node can also be selected as a forwarder, especially, in the case where the distance of DF to sink is comparatively greater than a node’s direct distance to sink. In such a scenario, the node itself will directly forward data to Sink. Neighbor node’s heat estimation will be performed likewise for Hot-Spot detection and avoidance. 

In order to find an increase in the temperature of human tissue upon exposure to RF communication, there is a need to calculate the SAR value of the tissue where the sensor is implanted. Equation (4) is used to calculate SAR. Temperature rise is estimated by using Equation (5) [10,31]:

(5) $$\Delta T=\left(SAR\right)\frac{t}{C}$$

where, t is the time duration for which tissue is exposed to electromagnetic radiation and C is the specific heat capacity.

The TAEO protocol also controls the energy utilization to increase the network lifetime and stability period. It first specifies a distance value. Afterward, it calculates the distance in between six non-critical nodes and to sink. During transmission, the distance between the two nodes involved in the communication is compared to the specified distance value. If it is below the defined distance limit, it means that the two nodes are closely placed. Therefore, it utilizes less transmission power. Otherwise, for distant nodes, the packet is transported with maximum energy.

### 4.3. Scheduling

Data submission to the DF node or to sink is TDMA based, in which a time slot is assigned to each node, during which it can submit the recorded information to the DF. If a node has no data to transmit during the assigned time slot, it declares itself as an idle node and moves to a standby state to save power.

## 5. Results

In this section, a comparative analysis is conducted that is based on analytical simulations of two state of the art protocols with the proposed protocol. The major performance parameters selected in this study are:

1. Throughput—No of packets successfully received at the sink.
2. Stability Period—Duration before the energy of the first node depletes completely and node dies.
3. Network Lifetime—Total time duration till the last node’s energy drains completely and all nodes in a network died.
4. Thermal Dissipation—Heat energy generated by the internal sensor circuitry and due to electromagnetic signals. This needs to be controlled for a hazard-free deployment of WBAN.

The evaluation of TAEO routing protocol is carried in comparison to ATTEMPT (Adaptive Threshold-based Thermal-aware Energy-efficient Multi-hop ProTocol) [19] and SIMPLE (Stable increased-throughput multi-hop protocol for link efficiency in wireless body area networks) [32].

### 5.1. Network Architecture

Eight miniature sensors are implanted in-body and a sink node is placed near its waist. The sensors read data from the body under observation related to temperature, heart rate, glucose, etc. Table 2 shows a list of sensors with the specified location. Data from heart rate and glucose monitoring devices are specified as critical and they are directly transferred to the sink. Also, all other sensor nodes will follow the normal multi-hop transmission. Sink will receive information from the critical and non-critical sensors and transmits it to the remote server through the internet. All of the nodes are equivalent in terms of energy consumption and processing power. When considering multihop transmissions, body movements may bring changes in topology. Table 2 depicts the initialization state of the network. Additionally, Table 3 elaborates on the simulation parameters. It also shows the SAR values and corresponding temperature change estimates [10,33].

### 5.2. Stability Period

**Figure 3:**
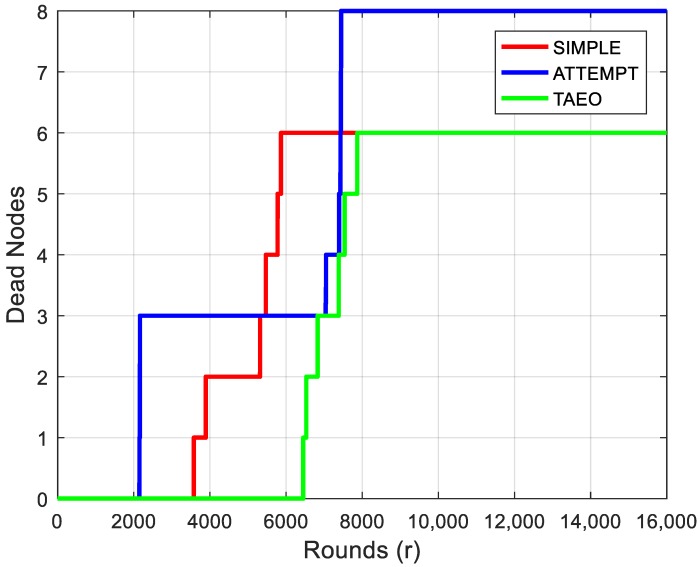
Evaluation of Network Lifetime & Stability Period.

Figure 3 represents the stability period analysis and proposed protocol supersedes when considering the stability period in comparison with ATTEMPT and SIMPLE protocols. Effective DF selection in each round along with hotspot node avoidance involves major elements that make proposed routing protocol more efficient. When comparing the stability period of three protocols, the first node in the proposed routing protocol dies in 6000th round, showing 50% improvement as compared to ATTEMPT and 28% with respect to the SIMPLE protocol. 

### 5.3. Residual Energy

**Figure 4:**
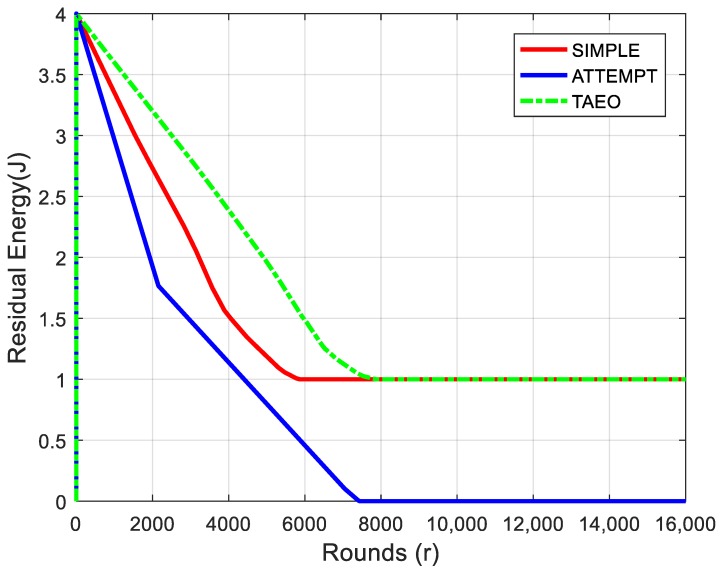
Energy Utilization.

Improvement in stability period also depicts the optimum power consumption of nodes in each round, which is illustrated in Figure 4 under the caption of residual energy. The reason behind this improvement is the careful selection of DF and, more importantly, the use of multiple transmission power levels with respect to distance between nodes. The results in Figure 4 depict that, due to better stability period, the nodes of TAEO consume less energy and significant improvement is observed. Sensor nodes with underlying ATTEMPT lost more than half of its energy until 2000th round, whereas the proposed routing protocol utilized a similar amount of energy in approximately 6000 rounds and SIMPLE scheme until 4000 rounds. 

### 5.4. Throughput

**Figure 5:**
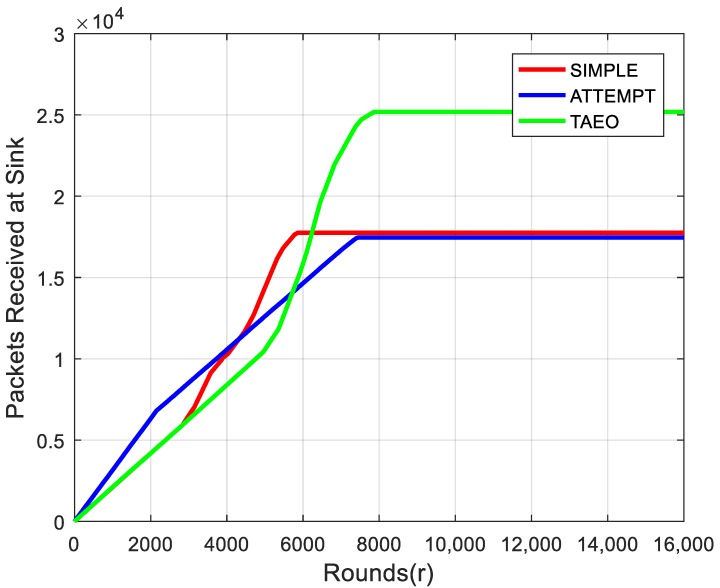
Throughput Evaluation.

Throughput is one of the major performance metrics in terms of routing protocols. Figure 5 shows that an average number of successful packets reached the sink. The optimized use of energy keeps the node alive for a longer period resulting in better throughput with respect to SIMPLE and ATTEMPT. Initially, until 6000 rounds, TAEO is not performing optimally in terms of throughput, as the proposed protocol tries to avoid heat generation due to rapid transmissions of nodes, however, after 6000 rounds, it performs better, as it was efficient in residual energy prolonging its network lifetime along with maximizing the number of packets reached at sink node, as can be seen in Figure 5.

### 5.5. Thermal Dissipation

**Figure 6:**
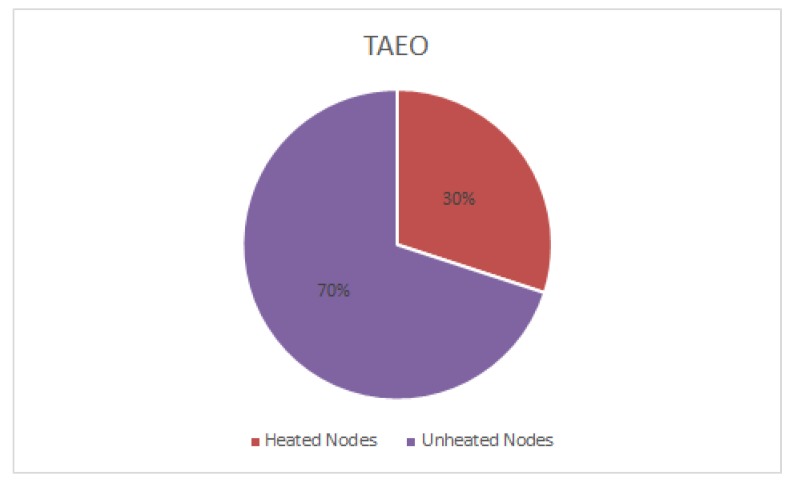
Heated Vs Unheated Nodes.

Figure 6 shows the ratio of the heated and unheated nodes after 16,000 rounds. The proposed protocol results in 70% saving i.e. only 30% of the total number of nodes become heated. Avoiding those implantable nodes in the routing path that have been heavily utilized and become heated does this. This is in contrast with the existing protocols, in which case 100% of the nodes become heated. 

## 6. Conclusions

The proposed protocol, TAEO, is a thermal dissipation controlled and highly stable routing protocol for WBAN. The selection of DF is based on node’s heat value and residual energy. It has shown significant improvements in stability period and overall lifetime of the network. The detection of a hot spot node is carried out by estimating the temperature rise of a node in each round. In the case that the temperature exceeds the specified threshold, it suspends the node for the coming few rounds until its temperature is restored to normal. Varying the levels of energy and decision for the amount of energy required for transmission based on the distance between two nodes pursuing transmission also optimizes power consumption by a node. Our simulation results show that the optimum utilization of energy for transmission has significantly enhanced the overall network lifetime and throughput of the network.

## Figures and Tables

**Table 1:** Related Work.

WBAN Model	Year	Methodology	Power Consumption	Packet
TARA	2005	Estimating temperature rise by monitoring the traffic level of neighbor nodes	High	Loss
LTR	2006	Node temperature is estimated by the observed neighbor node. Packet forwarded to the coolest neighbor	Medium	High
ALTR	2006	Forwarding of packets based on adaptive hop count	Medium	Low
LTRT	2007	Temperature is treated as the weight of node and least total weight route is adopted	Medium	Low
HPR	2007	Routes determined to apply the shortest hop algorithm. The packet is rerouted to the coolest neighbor in case of a hot-spot	High	Low
THSR	2009	Like HPR, except two thresholds are used.	High	Low
M-ATTEMPT	2013	Hot-Spot node detection and suitable low-temperature path selection	Low	Low
M^2^E^2^	2014	A multi-mode scheme, at home it uses the underlying home network for transmission using single-hop, otherwise, nodes on body maintain a routing table and use conventional multi-hop method for transmission	Low	Low

**Table 2:** Sensor Nodes Deployment.

Sensor Location	X-Coordinate	Y-Coordinate
Waist (Sink)	0.25	1
Knee	0.5	0.3
Calf	0.3	0.1
Left Thigh	0.3	0.55
Right Thigh	0.5	0.55
Glucose (Critical)	0.37	0.75
Heart Rate (Critical)	0.45	0.9
Left Palm	0.7	0.8
Right Palm	0.1	0.8

**Table 3:** Simulation Parameters.

Tissue Type	Frequency	Thermal Conductivity	Electric Field	Density	SAR	Temp
Nerve	800	0.16	38.89	1040	0.232	0.022
	850	0.17	40.08	1040	0.262	0.025
	900	1.8	41.25	1040	0.294	0.028
